# Stroma AReactive Invasion Front Areas (SARIFA), tumour immune microenvironment, and survival in colorectal cancer

**DOI:** 10.1038/s41416-025-02972-z

**Published:** 2025-03-07

**Authors:** Vilja V. Tapiainen, Päivi Sirniö, Hanna Elomaa, Henna Karjalainen, Ville K. Äijälä, Meeri Kastinen, Akseli Kehusmaa, Vesa-Matti Pohjanen, Outi Lindgren, Onni Sirkiä, Maarit Ahtiainen, Olli Helminen, Erkki-Ville Wirta, Jukka Rintala, Juha Saarnio, Tero Rautio, Toni T. Seppälä, Jan Böhm, Jukka-Pekka Mecklin, Anne Tuomisto, Markus J. Mäkinen, Juha P. Väyrynen

**Affiliations:** 1 Translational Medicine Research Unit, Medical Research Center Oulu, Oulu University Hospital, and University of Oulu, Oulu, Finland; 2 Department of Biological and Environmental Science, University of Jyväskylä, Jyväskylä, Finland; 3 Department of Education and Research, Hospital Nova of Central Finland, Well Being Services County of Central Finland, Jyväskylä, Finland; 4 Department of Pathology, Hospital Nova of Central Finland, Well Being Services County of Central Finland, Jyväskylä, Finland; 5 Department of Environmental and Biological Sciences, University of Eastern Finland, Kuopio, Finland; 6 Central Finland Biobank, Hospital Nova of Central Finland, Well Being Services County of Central Finland, Jyväskylä, Finland; 7 Department of Gastroenterology and Alimentary Tract Surgery, Tampere University Hospital, Tampere, Finland; 8 Faculty of Medicine and Health Technology, Tampere University and Tays Cancer Centre, Tampere University Hospital, Tampere, Finland; 9 Department of Gastrointestinal Surgery, Helsinki University Central Hospital, University of Helsinki, Helsinki, Finland; 10 Applied Tumour Genomics, Research Program Unit, University of Helsinki, Helsinki, Finland; 11 Faculty of Sport and Health Sciences, University of Jyväskylä, Jyväskylä, Finland

**Keywords:** Colorectal cancer, Cancer microenvironment

## Abstract

**Background:**

SARIFA (Stroma AReactive Invasion Front Areas), defined as the direct contact between a tumour cell cluster and adipose cells at the invasion margin, has been proposed as a prognostic marker in gastrointestinal cancers. We hypothesized that SARIFA is associated with an immunosuppressive tumour microenvironment.

**Methods:**

SARIFA status was evaluated in two large colorectal cancer cohorts (*N* = 1876). Survival analyses were performed using multivariable Cox regression. Immune cell densities were analysed utilizing multiplex and conventional immunohistochemistry combined with digital image analysis.

**Results:**

SARIFA-positivity was independently associated with a shorter cancer-specific survival in both cohorts [Cohort 1: hazard ratio (HR) for SARIFA-positive (vs. negative) 1.75 (95% CI 1.35–2.25), *P* < 0.0001; Cohort 2: HR for SARIFA-positive (vs. negative) 2.09 (95% CI 1.43–3.05), *P* = 0.0001]. SARIFA-positivity was associated with lower densities of CD3^+^ T cells, CD66b^+^ granulocytes, M1-like macrophages, and CD14^+^HLA-DR^+^ mature monocytic cells, but higher densities of M2-like macrophages and CD14^+^HLA-DR^-^ immature monocytic cells. Mean Cohen’s kappa for SARIFA evaluation between eight investigators was 0.80.

**Conclusions:**

SARIFA status is a highly reproducible, independent prognostic factor in colorectal cancer. SARIFA-positivity is associated with lower densities of antitumourigenic immune cells and the polarisation of macrophages towards a protumourigenic M2-like phenotype.

## Introduction

Colorectal cancer is the third most diagnosed cancer with over 1.9 million new cases a year [1]. The tumour microenvironment is a dynamic, heterogeneous mix of noncellular and cellular components [2, 3]. The surrounding cells constantly interact with cancer cells, inducing both tumour promoting and tumour suppressing functions that affect patient survival [3, 4]. Colorectal cancers are prognostically classified by the TNM staging system, which describes the extent of cancer spread without assessing the characteristics of the tumour microenvironment [5]. However, the clinical outcomes may vary within the same disease stage [6–8]. Therefore, additional histomorphological and immunological factors, such as tumour budding and Immunoscore, are needed for more detailed prognostic classification and targeted treatment [8, 9].

Stroma AReactive Invasion Front Areas (SARIFA), first discovered in 2021, is a prognostic factor for colorectal, gastric, and oesophageal cancer, which can be assessed using haematoxylin & eosin (H&E)-stained slides [10–12]. SARIFA-positivity is defined as the direct contact between a tumour gland/tumour cell cluster (≥5 cells) and adipose tissue at the invasion front [10]. The prognostic power of SARIFA has been suggested to be based on tumour-adipocyte interaction, potentially including an altered immune response [13, 14]. SARIFA has not been associated with distinct genetic alterations [15]. The prognostic power of SARIFA has not yet been validated by an independent research group. Moreover, further studies on the immune responses associated with SARIFA might enlighten the mechanisms behind the prognostic value of SARIFA.

Here, we set out to examine the prognostic significance of SARIFA and immunological features associated with it in two large cohorts of patients with colorectal cancer (*N* = 1876).

## Methods

### Study design and study population

In this cohort study, two independent cohorts of patients with colorectal cancer were analysed. The cohorts included surgically treated stage I-IV colorectal cancer patients from whom adequate tumour samples were available. Cohort 1 was retrospectively collected from patients (*N* = 1343) operated on at Central Finland Central Hospital in 2000–2015 [16]. Cohort 2 was prospectively collected at Oulu University Hospital starting from 2006. It has been previously described from 2006 to 2014 [17, 18] and has now been extended until 2020, consisting of 1011 patients. The patients who had received preoperative radiotherapy or chemoradiotherapy were excluded from further analyses (Cohort 1, *N* = 243; Cohort 2, *N* = 235), due to morphological changes of tumours associated with preoperative treatments. After the exclusion, there were 1100 patients for cohort 1 and 776 patients for cohort 2.

In survival analyses, the patients who had died in less than 30 days from the surgery were additionally excluded (cohort 1, *N* = 37; cohort 2, *N* = 5), resulting in 1063 patients for cohort 1 and 771 for cohort 2. (Supplementary Fig. S1) The study endpoints were overall survival (time from operation to death or end of follow-up) and colorectal cancer-specific survival (time from operation to colorectal cancer death or end of follow-up). Follow-up time was limited to 10 years, considering that most colorectal cancer deaths occur within that time. During the follow-up, there were 531 deaths of which 296 were colorectal cancer deaths in cohort 1. In cohort 2 there were 284 deaths of which 155 were colorectal cancer deaths. The median follow-up time for censored cases was 10 years (IQR 7.3–10) in cohort 1 and 7.0 years (IQR 4.7–10) in cohort 2.

### Histopathologic analysis

Histological samples were fixed using 10% formalin, embedded in paraffin, and H&E stained. H&E-stained slides were scanned with either Hamamatsu (NanoZoomer S60 or NanoZoomer-XR) or Leica Aperio AT2 slide scanner and assessed by digital microscopy (NDP.view2 or Aperio ImageScope). Basic patient and tumour characteristics, such as TNM-stage and sex, were previously collected in both cohorts [16–18]. Tumour budding was assessed following ITBCC criteria [9] by a researcher (VKÄ) with expertise in colorectal cancer histopathology. Tumour differentiation was evaluated according to WHO 2019 criteria by a gastrointestinal pathologist (JPV, cohort 1) or VKÄ (cohort 2). Interobserver agreement between VKÄ and JPV was investigated using 30 consecutive cases from cohort 1, yielding kappa scores of 0.70 and 0.71 for tumour budding and tumour grade, respectively. Lymphovascular invasion, defined as tumour cells within vascular spaces, was assessed by JPV using H&E-stained sections. Immunohistochemistry was used to determine MMR status and *BRAF* V600E mutation status [16, 19]. A previous study of 147 patients from cohort 2 indicated a sensitivity of 100% and specificity of 99.3% for *BRAF* V600E mutation specific immunohistochemistry in detecting *BRAF* mutation status, compared to sequencing [19].

**Fig. 1:**
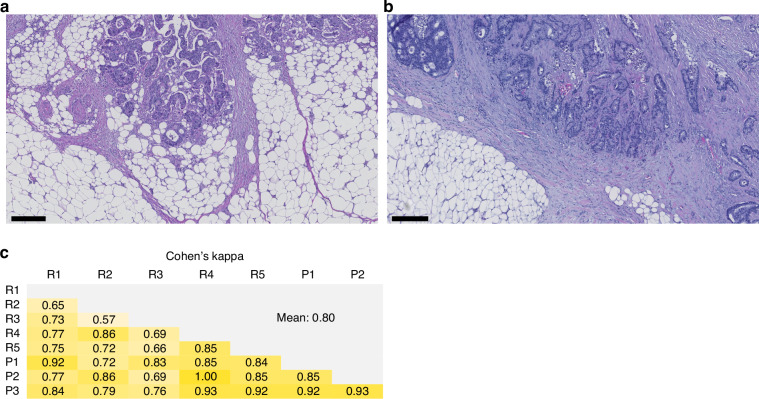
Evaluation of Stroma AReactive Invasion Front Areas (SARIFA) status in colorectal cancer. **a** A representative example of a SARIFA-positive case. **b** A representative example of a SARIFA-negative case. Scale bars are 250 µm. **c** The reproducibility of SARIFA status evaluation between eight investigators, measured with Cohen’s kappa. R researcher, P pathologist

SARIFA status was visually evaluated from images of H&E-stained whole slide sections according to the previously published criteria [10, 14]. In brief, SARIFA-positivity was defined as the direct contact between a tumour gland/tumour cell cluster (≥5 cells) and surrounding adipose tissue in the invasion front [10]. The case was classified as SARIFA-positive if there was at least one SARIFA-positive focus [10] (Fig. 1). The evaluation was performed by a researcher with expertise in colorectal cancer histopathology (VVT), blinded to the study endpoints. In cohort 1, SARIFA status was assessed using a single whole-slide image per case representing the deepest invasion. In cohort 2, SARIFA status was determined using an average of 3 whole-slide images per case (range 1–18). To evaluate the consistency of SARIFA status evaluation, we compared the assessment from a single slide section containing the deepest invasion area with that from multiple slide sections across 30 consecutive cases in cohort 2. The Cohen’s kappa value between these two approaches was 0.89, indicating high consistency between SARIFA status derived from a single tumour slide with the deepest invasion and that based on multiple slides. In 29 out of 30 consecutive cases (97%), SARIFA status corresponded to the slide representing the deepest invasion area, indicating that additional slides were needed in only 3% of cases to identify a SARIFA-positive area. For cohort 1, we additionally studied the locations of the SARIFA-positive foci (submucosa or subserosa).

The reproducibility of SARIFA status evaluation was tested by 8 examiners (three pathologists and five researchers) independently assessing SARIFA status of 30 cases.

In cohort 1, the histological depth of subserosal invasion in pT3/4 tumours was measured on H&E-stained whole slide images using the Ruler tool of the digital microscope (NDP.view2). Measurements followed previously published criteria [20], defining the depth as the distance between the last identifiable smooth muscle cell of the residual tunica muscularis propria and the cancer cell with the deepest localisation within the pericolic/perirectal fat. If the tunica muscularis propria was completely destroyed, measurements were taken from the first identifiable pericolic/perirectal fat cell. The cut-off value of 3.00 mm was based on the previously published criteria [20].

### Immune cell analyses

Immune cell densities were determined using tissue microarrays consisting of tissue cores of 1 mm diameter, with an aim to include 2 cores from the tumour centre and 2 cores from the invasive margin [16]. Multiplex immunohistochemistry combined with digital image analysis was utilized to evaluate the immune cell densities of tumour samples for cohort 1. The three assays were based on a cyclic method with AEC as the chromogen, described and validated earlier in detail [21, 22]. The multiplex immunohistochemistry protocol and antibodies included in the assays are presented in Supplementary Fig. S2. Image analysis was conducted using machine learning algorithms in QuPath [23], an open source software package for digital pathology. The process involved training random forest pixel classifiers to identify tumour epithelial and stromal regions for analysis and random forest object classifiers to classify cell based on shape, intensity, and texture features [21, 22]. The cell types analysed in this study included CD3^+^ T cells, CD20^+^CD79A^+^ B cells, CD20^-^CD79A^+^ plasma cells, CD68^+^/CD163^+^ macrophages, M1-like, M2-like macrophages, CD14^+^ monocytic cells, CD14^+^HLA-DR^+^ mature monocytic cells, CD14^+^HLA-DR^-^ immature monocytic cells, CD66b^+^ granulocytes, and tryptase^+^ mast cells. M1-like and M2-like macrophages were defined according to their *macrophage polarization index* [(CD86 + HLADR)-(CD163 + MRC1), with marker names denoting intensity percentiles across all cases] [21]. The immune cell data from the three multiplex immunohistochemistry assays were analysed as cell densities per mm^2^ and were not categorised for the analyses. Conventional immunohistochemistry combined with digital image analysis was utilized to determine CD3^+^ T cell and CD8^+^ T cell densities for cohorts 1 and 2 [16], and these densities were used to evaluate Immune cell score following the principles of the Immunoscore [16, 24]. Multiplex immunohistochemistry data were available for 1065 patients (T cells and macrophages), 1070 patients (B cells and plasma cells), and 1045 patients (monocytic cells, granulocytes, and mast cells), while single-colour immunohistochemistry data for CD3 and CD8 were available for 1017 patients in cohort 1 and 751 patients in cohort 2. In addition, we performed subgroup analyses of multiplex immunohistochemistry data consisting of cases with both SARIFA-positive and SARIFA-negative invasion margin cores (*N* = 49) (Supplementary Fig. S3).

### Statistical analyses

Statistical analyses were carried out using IBM SPSS Statistics for Windows (IBM Corp. version 29.0). *P*-value of <0.05 was considered statistically significant.

Cross-tabulation and Chi-square test were used to analyse SARIFA status in relation to tumour and patient characteristics. Immune cell densities were reported as median values and interquartile ranges or presented as boxplots. Statistical significance of the associations between SARIFA status and immune cells were determined by using Mann-Whitney *U* test. Tumour and patient characteristics and survival analyses were also performed in pT3/T4 patient subgroup, considering that most SARIFA-positive cases occur in pT3/4 patients, as SARIFA is usually observed at subserosal fat. The reproducibility of SARIFA evaluation was examined using Cohen’s kappa coefficients.

Cox proportional hazards regression models and Kaplan–Meier estimates were used to investigate SARIFA status in relation to cancer-specific survival and overall survival. Multivariable Cox regression models were adjusted for age (<65, 65–75, >75), sex (male, female), year of operation (Cohort 1: 2000–2005, 2006–2010, 2011–2015 and Cohort 2: 2006–2010, 2011–2015, 2016–2020), tumour location (proximal colon, distal colon, rectum), AJCC disease stage (I-II, III, IV), tumour grade (low-grade, high-grade), lymphovascular invasion (no, yes), MMR status (proficient, deficient), *BRAF* mutation (wild-type, mutant), and tumour budding (grade 1, 2, 3). For multivariable models, missing data (*BRAF* status: 2 patients in cohort 1, 7 patients in cohort 2) were included in the majority category (*BRAF* wild type), to limit the degrees of freedom.

## Results

### Tumour and patient characteristics

We first analysed the associations of SARIFA status with tumour and patient characteristics for 1100 patients in cohort 1 and 776 patients in cohort 2 (Table 1), as well as 875 patients with pT3/4 tumours from cohort 1 and 547 patients with pT3/4 tumours from cohort 2 (Supplementary Table S1). Most of the SARIFA-positive cases belonged to the pT3/4 group, with the remaining cases (6 in cohort 1) showing SARIFA-positivity in the submucosa. In the group of all patients, 326 (30%) patients in cohort 1 and 243 (31%) patients in cohort 2 were classified as SARIFA-positive. Among the SARIFA-positive cases in cohort 1, the SARIFA-positive focus was identified in the subserosal fat in 294 (90%) cases, in the submucosa in 6 (2%) cases, and in both regions in 26 (8%) cases. In the pT3/4 subgroup, 320 (37%) patients in cohort 1 and 243 (44%) patients in cohort 2 were classified as SARIFA-positive. In the group of all patients, SARIFA-positivity was significantly associated with tumour location in the colon, high disease stage, high tumour grade, lymphovascular invasion, high tumour budding grade, and low immune cell score in both cohorts (Table 1, *P* < 0.001 for all comparisons). SARIFA-positivity was associated with *BRAF* mutation in cohort 1 (*P* = 0.026), but not significantly in cohort 2 (*P* = 0.072). SARIFA status was not associated with MMR status. SARIFA-positivity was significantly associated with deeper histological depth of subserosal invasion of pT3/4 tumours (Supplementary Table S1, *P* < 0.0001).

**Table 1:** Baseline characteristics of colorectal cancer patients according to SARIFA status.

	Cohort 1				Cohort 2			
		SARIFA status				SARIFA status		
Characteristic	Total N	Negative	Positive	*P*	Total N	Negative	Positive	*P*
All cases	1100 (100%)	774 (70%)	326 (30%)		776 (100%)	533 (69%)	243 (31%)	
Sex								
Female	557 (51%)	338 (50%)	169 (52%)	0.64	364 (47%)	244 (46%)	120 (49%)	0.35
Male	543 (49%)	386 (50%)	157 (48%)		412 (53%)	289 (54%)	123 (51%)	
Age (years)								
<65	290 (26%)	189 (24%)	101 (31%)	0.075	233 (30%)	151 (28%)	82 (34%)	0.15
65–75	381 (35%)	277 (36%)	104 (32%)		285 (37%)	194 (36%)	91 (37%)	
>75	429 (39%)	308 (40%)	121 (37%)		258 (33%)	188 (35%)	70 (29%)	
Year of operation								
2000–2005	342 (31%)	240 (31%)	102 (31%)	0.57	–	–	–	0.10
2006–2010	353 (32%)	242 (31%)	111 (34%)		155 (20%)	98 (18%)	57 (23%)	
2011–2015	405 (37%)	292 (38%)	113 (35%)		218 (28%)	145 (27%)	73 (30%)	
2016–2020	–	–	–		403 (52%)	290 (54%)	113 (47%)	
Tumour location								
Proximal colon	536 (49%)	363 (47%)	173 (53%)	0.004	323 (42%)	205 (38%)	118 (49%)	<0.0001
Distal colon	404 (37%)	281 (36%)	123 (38%)		205 (26%)	127 (24%)	78 (32%)	
Rectum	160 (15%)	130 (17%)	30 (9%)		248 (32%)	201 (38%)	47 (19%)	
AJCC disease stage								
I	184 (17%)	181 (23%)	3 (1%)	<0.0001	187 (24%)	187 (35%)	0 (0%)	<0.0001
II	408 (37%)	327 (42%)	81 (25%)		253 (33%)	183 (34%)	70 (29%)	
III	355 (32%)	192 (25%)	163 (50%)		251 (32%)	131 (25%)	120 (49%)	
IV	153 (14%)	74 (10%)	79 (24%)		85 (11%)	32 (6%)	53 (22%)	
Tumour grade								
Low-grade	903 (82%)	667 (86%)	236 (72%)	<0.0001	665 (86%)	478 (90%)	187 (77%)	<0.0001
High-grade	197 (18%)	107 (14%)	90 (28%)		111 (14%)	55 (10%)	56 (23%)	
Lymphovascular invasion								
No	858 (78%)	667 (86%)	191 (59%)	<0.0001	429 (55%)	369 (69%)	60 (25%)	<0.0001
Yes	242 (22%)	107 (14%)	135 (41%)		347 (45%)	164 (31%)	183 (75%)	
Tumour budding								
Grade 1	827 (75%)	647 (84%)	180 (55%)	<0.0001	541 (70%)	439 (81%)	111 (46%)	<0.0001
Grade 2	156 (14%)	84 (11%)	72 (22%)		129 (17%)	59 (11%)	70 (29%)	
Grade 3	117 (11%)	43 (6%)	74 (23%)		106 (14%)	44 (8%)	62 (26%)	
MMR status								
MMR proficient	931 (85%)	652 (84%)	279 (86%)	0.59	652 (84%)	443 (83%)	209 (86%)	0.31
MMR deficient	169 (15%)	122 (16%)	47 (14%)		124 (16%)	90 (17%)	34 (14%)	
*BRAF* status^a^								
Wild-type	916 (83%)	657 (85%)	259 (79%)	0.026	662 (86%)	462 (88%)	200 (83%)	0.072
Mutant	182 (17%)	115 (15%)	67 (21%)		107 (14%)	65 (12%)	42 (17%)	
Immune cell score^b^								
Low	170 (17%)	107 (15%)	63 (21%)	<0.0001	114 (15%)	54 (11%)	60 (25%)	<0.0001
Intermediate	619 (61%)	421 (59%)	198 (65%)		432 (58%)	303 (59%)	129 (54%)	
High	228 (22%)	185 (26%)	43 (14%)		205 (27%)	156 (31%)	49 (21%)	

*P* values were calculated using the Chi-square test.

*AJCC* American Joint Committee on Cancer, *MMR* mismatch repair.

^a^Data missing for 2 cases in cohort 1 and 7 cases in cohort 2.

^b^Data missing for 83 cases in cohort 1 and 25 cases in cohort 2.

### Survival

**Fig. 2:**
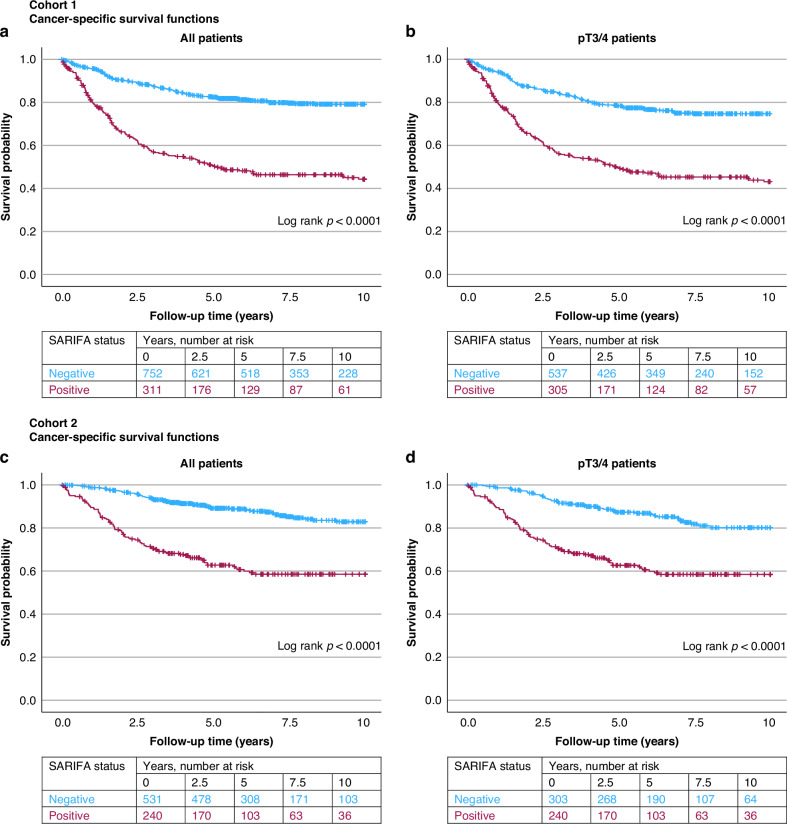
Stroma AReactive Invasion Front Areas (SARIFA) status and survival in colorectal cancer. Kaplan–Meier curves for cancer specific survival in cohort 1 (**a**, **b**) and in cohort 2 (**c**, **d**). All patients (**a**, **b**) and the pT3/4 patient subgroup (**b**, **d**) were analysed.

In Kaplan–Meier analysis, SARIFA status was associated with shorter ten-year cancer-specific survival in all patients and in the pT3/4 subgroup in both cohorts (Log rank *P* < 0.0001) (Fig. 2).

Univariable and multivariable Cox regression models showed that SARIFA status was significantly associated with cancer-specific survival and overall survival in all patients and in the pT3/T4 subgroup in both cohorts (Table 2, Supplementary Table S2). The multivariable HRs for colorectal cancer death in SARIFA-positive patients of cohort 1 were for all patients 1.75 (95% CI: 1.35–2.25, *P* < 0.0001) and for pT3/T4 patients 1.62 (95% CI 1.25–2.10, *P* = 0.0002). The multivariable HRs for colorectal cancer death in SARIFA-positive patients of cohort 2 were for all patients 2.09 (95% CI 1.43–3.05, *P* = 0.0001) and for pT3/T4 patients 2.26 (95% CI 1.50–3.42, *P* = 0.0001). The multivariable models were adjusted for common prognostic factors, such as disease stage, lymphovascular invasion, tumour budding, MMR status, and *BRAF* status. A direct comparison of the prognostic power of SARIFA status and tumour budding using Cox regression models for cancer-specific survival is presented in Supplementary Table S3.

**Table 2:** Univariable and multivariable Cox regression models for cancer-specific survival and overall survival according to SARIFA status in colorectal cancer.

		Colorectal cancer-specific survival			Overall survival		
Variable	No. of cases	No. of events	Univariable HR (95% CI)	Multivariable HR (95% CI)	No. of events	Univariable HR (95% CI)	Multivariable HR (95% CI)
Cohort 1							
SARIFA status, all tumours							
Negative	752	140	1 (referent)	1 (referent)	328	1 (referent)	1 (referent)
Positive	311	156	3.56 (2.83–4.48)	1.75 (1.35–2.25)	203	2.05 (1.72–2.44)	1.58 (1.30–1.92)
*P*			<0.0001	<0.0001		<0.0001	<0.0001
SARIFA status, pT3/4							
Negative	537	122	1 (referent)	1 (referent)	256	1 (referent)	1 (referent)
Positive	305	156	2.89 (2.28–3.67)	1.62 (1.25–2.10)	202	1.84 (1.53–2.21)	1.46 (1.20–1.79)
*P*			<0.0001	0.0002		<0.0001	0.0002
Cohort 2							
SARIFA status, all tumours							
Negative	531	65	1 (referent)	1 (referent)	170	1 (referent)	1 (referent)
Positive	240	90	3.72 (2.70–5.12)	2.09 (1.43–3.05)	114	1.83 (1.44–2.32)	1.48 (1.12–1.95)
*P*			<0.0001	0.0001		<0.0001	0.006
SARIFA status, pT3/4							
Negative	303	45	1 (referent)	1 (referent)	104	1 (referent)	1 (referent)
Positive	240	90	3.09 (2.16–4.42)	2.26 (1.50–3.42)	114	1.71 (1.32–2.25)	1.50 (1.11–2.03)
*P*			<0.0001	0.0001		<0.0001	0.008

Multivariable Cox proportional hazards regression models were adjusted for sex, age (<65, 65–75, >75), year of operation (Cohort 1: 2000–2005, 2006–2010, 2011–2015 and Cohort 2: 2006–2010, 2011–2015, 2016–2020), tumour location (proximal colon, distal colon, rectum), disease stage (I-II, III, IV), tumour grade (low-grade, high-grade), lymphovascular invasion (negative, positive), tumour budding (grade 1, grade 2, grade 3), mismatch repair (MMR) status (proficient, deficient) and *BRAF* status (wild-type, mutant).

*CI* confidence interval, *HR* hazard ratio.

### Immune cells

**Fig. 3:**
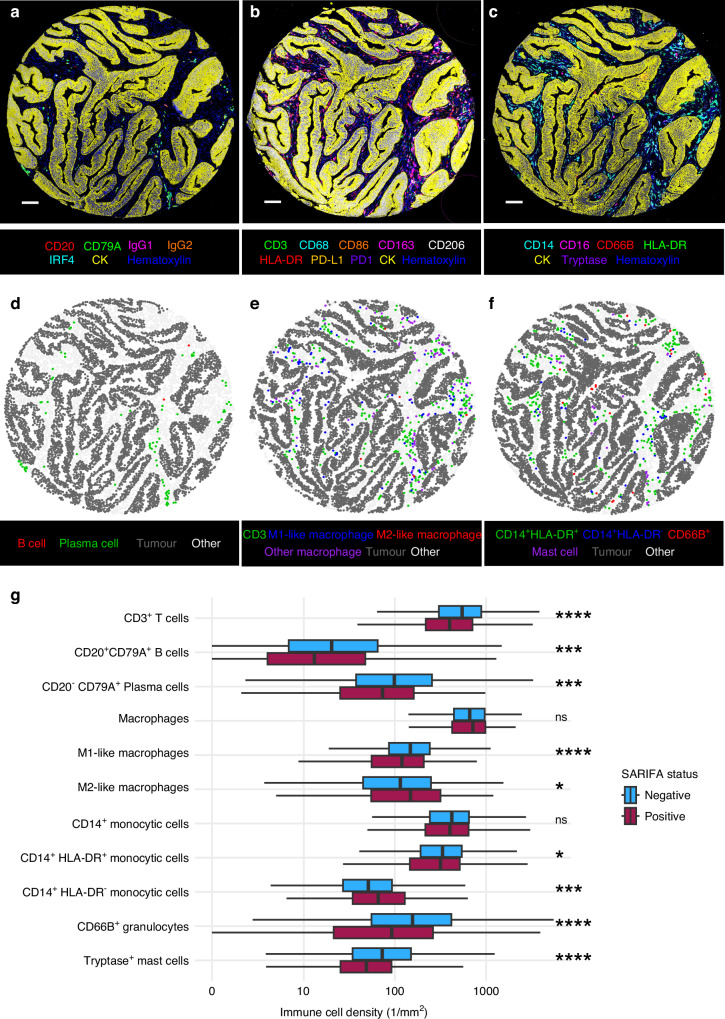
Multiplex immunohistochemistry panel, image analyses and immune cell densities. Example images of multiplex immunohistochemistry for detecting B cells (**a**), macrophages (**b**) and myeloid cells (**c**). Scale bars are 100 µm. Digital image analysis was utilized to create corresponding cell maps (**d**–**f**). Boxplot of immune cell densities (**g**) according to SARIFA status in colorectal cancer. Analyses were based on cohort 1 immune cell data, in which *N* = 1065 for CD3^+^ T cells, macrophages, M1-like macrophages, and M2-like macrophages; *N* = 1070 for CD20^+^CD79A^+^ B cells and CD20^-^CD79A^+^ plasma cells; *N* = 1045 for CD14^+^ monocytic cells, CD14^+^HLA-DR^+^ mature monocytic cells, CD14^+^HLA-DR^-^ immature monocytic cells, CD66B^+^ granulocytes, and tryptase^+^ mast cells. ns: *p* > 0.05, *p < 0.05, **p < 0.01, ***p < 0.001. ****p < 0.0001.

Immune cells were assessed with multiplex immunohistochemistry combined with digital image analysis in cohort 1 (Fig. 3). SARIFA-positivity was associated with lower densities of CD3^+^ T cells (*P* < 0.0001), CD66b^+^ granulocytes (*P* < 0.0001), Tryptase^+^ mast cells (*P* < 0.0001), CD20^+^CD79A^+^ B cells (*P* < 0.001), and CD20^-^CD79A^+^ plasma cells (*P* < 0.001).

SARIFA-positivity was not significantly associated with the overall density of macrophages. However, SARIFA-positivity was associated with lower density of M1-like macrophages (*P* < 0.0001) and higher density of M2-like macrophages (*P* < 0.05). SARIFA-positivity was not significantly associated with the overall density of CD14^+^ monocytic cells, but SARIFA-positivity was associated with lower density of CD14^+^HLA-DR^+^ mature monocytic cells (*P* < 0.05) and higher density of CD14^+^ HLA-DR^-^ immature monocytic cells (*P* < 0.001).

The association of SARIFA-positivity with lower T cell densities was confirmed with conventional immunohistochemistry in cohort 2 (Supplementary Table S4).

To assess whether immune infiltrates are heterogeneous within SARIFA-positive cases, we performed a subgroup analysis of cases with both SARIFA-positive and SARIFA-negative TMA cores from the invasive margin. In this analysis, the only statistically significant difference was a tendency for higher M1-like macrophage densities in SARIFA-positive cores compared to SARIFA-negative cores (*P* < 0.05) (Supplementary Fig. S3).

The prognostic power of immune cell score and SARIFA was compared using Cox regression models for cancer-specific survival (Supplementary Table S5). In multivariable models adjusted for common prognostic factors, the HRs for the high immune cell score were 0.55 (95% CI: 0.35–0.85, *P*_trend_ = 0.006) in cohort 1 and 0.54 (95% CI: 0.30–1.00, *P*_trend_ = 0.037) in cohort 2, while the HRs for SARIFA-positivity were 1.71 (95% CI: 1.32–2.23, *P* < 0.0001) in cohort 1 and 1.86 (95% CI: 1.26–2.75, *P* = 0.002) in cohort 2.

### Reproducibility

The reproducibility of SARIFA evaluation was tested in 30 tumours by eight examiners. Mean Cohen’s kappa between the examiners was 0.80, representing substantial agreement (Fig. 1).

## Discussion

In this large study that included 1876 patients with colorectal cancer, SARIFA status was an independent prognostic factor for cancer-specific survival and overall survival. SARIFA can be reproducibly evaluated using H&E-stained tumour sections. Furthermore, an immune suppressed microenvironment, characterized by lower densities of antitumourigenic immune cells but higher densities of M2-like macrophages and immature monocytic cells, was demonstrated in SARIFA-positive cases. These findings validate SARIFA as a useful prognostic parameter in colorectal cancer, with prognostic significance comparable or superior to several established markers, and highlight the interplay between SARIFA and the tumour immune microenvironment.

We found that SARIFA-positivity strongly associated with unfavourable disease outcome. In a few previous studies, SARIFA status has been associated with shorter overall, disease-specific, and progression free survival in colorectal cancer [10, 15] and with shorter overall survival in gastric [12, 13] and oesophagogastric cancer [11]. Our study is, to our knowledge, the largest so far and provides confirmation of the prognostic power of SARIFA by an independent research group in two large cohorts. Moreover, the study benefited from the extensive inclusion of additional prognostic parameters in the multivariable survival models. In our study, the significance of SARIFA exceeded several clinically relevant prognostic factors, including tumour budding [9], lymphovascular invasion, and MMR status [25]. Routine reporting of tumour budding is advocated in clinical guidelines [9, 26, 27]. Therefore, SARIFA could potentially have value in clinical practice due to its high prognostic relevance, low inter-observer variability, and fast determination on H&E slides. Further studies should compare the relative significance of SARIFA and tumour budding in various patient subgroups.

We analysed immune cell densities according to SARIFA status using multiplex immunohistochemistry in cohort 1. SARIFA-positivity was mostly associated with lower immune cell densities, including T cells, CD66b^+^ granulocytes, and mast cells. The finding for T cells was also confirmed using conventional immunohistochemistry in cohort 2. In a previous study, the associations of SARIFA status with peripheral blood lymphocytes were studied using flow cytometry, and there were no differences in the frequency of most lymphocyte populations [14]. In that study, there were also no differences in CD3^+^ and CD8^+^ T cell densities of tumour samples between SARIFA-positive and SARIFA-negative cases [14]. The difference between that study and ours may be related to the lower sample size in the previous study (*N* = 45), as well as the differences in immune cell analysis methodology. We utilized a well-validated machine learning based image analysis method combined with multiplex/conventional immunohistochemistry in our analyses [16, 21, 22]. However, in the previous study, SARIFA-positive cases had significantly less natural killer cells in peripheral blood and in the tumour microenvironment [14]. SARIFA-positive cases have also been associated with lower expression of IL6 and TNFA in gastric cancer [13]. The depletion of these proinflammatory cytokines may account for the association that we observed between SARIFA-positivity and lower immune cell densities.

The multiplexed method that we employed also enabled us to analyse macrophage polarisation and myeloid cell maturation in a manner not possible using conventional single-plex immunohistochemistry [28, 29]. For example, macrophage polarisation was analysed by calculating polarisation indices based on the expression levels of four polarisation markers (CD86, HLADR, CD163, MRC1) at single cell resolution. Macrophages exist in a spectrum of polarisation states of which M1-like macrophages are classically activated and proinflammatory, while M2-like macrophages are alternatively activated and anti-inflammatory [30]. These polarisation states cannot be reliably captured by a single polarisation marker. SARIFA status was associated with the polarisation of macrophages; while the overall densities of macrophages remained unchanged, SARIFA-positive cases were associated with lower densities of M1-like macrophages and higher densities of M2-like macrophages compared to SARIFA-negative cases. However, in subgroup analyses of cases with both SARIFA-positive and SARIFA-negative TMA cores from the invasive margin, the only significant finding was a tendency for higher M1-like macrophage densities in SARIFA-positive cores. This suggests that the immune cell differences between SARIFA-positive and SARIFA-negative cases may reflect a broader effect rather than localized immune cell changes in SARIFA-positive regions. However, the limited sample size of this subgroup analysis may also have influenced the finding. In previous studies, SARIFA-positivity has been associated with increased macrophage (CD68+) infiltration at the invasive margin of gastric cancer [12]. Tumour-associated macrophages are frequently shifted towards an M2-like phenotype [31] that can induce pro-tumoural effects helping the tumour cells to evade immune system and spread to other organs [32]. High densities of M2-like macrophages have been associated with worse prognosis in colorectal cancer [33, 34] and higher densities of M1-like macrophages have been associated with favourable prognosis [34].

Our results of myeloid cell maturation showed that SARIFA-positivity was associated with lower densities of CD14^+^HLA-DR^+^ mature monocytic cells and higher densities of CD14^+^ HLA-DR^-^ immature monocytic cells. These immature monocytic cells could include myeloid-derived suppressor cells, although their definite demonstration would require functional suppression assays that are not compatible with formalin-fixed paraffin embedded material [29]. Myeloid-derived suppressor cells are pathologically activated immature myeloid cells that have been converted into immunosuppressive cells by tumour-related inflammation signals [35, 36]. They may promote tumour progression by stimulating an immunosuppressive microenvironment, angiogenesis, and formation of metastasis [29, 36, 37].

Previous studies have hypothesized that the mechanisms behind the prognostic significance of SARIFA can be explained by a lipid-mediated immunosuppressive TME in SARIFA-positive patients [13]. Several lipid-driven pathways may promote M2-like macrophages [38]. SARIFA-positivity has also been associated with the upregulation of fatty acid metabolism [12, 15], including the upregulation of FABP4 and CD36 in gastric cancer [12]. Our findings of the altered immune cell densities support the hypothesis of immunosuppressive microenvironment and the polarisation of macrophages towards M2-phenotype in SARIFA-positive cases, and this may be related to a lipid-mediated immunosuppressive TME.

Our study has some limitations to consider. First, the analyses of cohort 1 were conducted using a single whole-slide image, containing the area of deepest tumour invasion. More broadly, the sampling of tumours may also limit the representativeness of the invasive margin in which SARIFA status can be assessed. However, our validation analysis suggests that SARIFA-positivity is most likely present at the deepest invasive margin. This region is typically included in the histological samples, as it is needed for accurate pT category assessment in TNM classification. Nonetheless, SARIFA cannot be reliably assessed from preoperative biopsies, as the deep invasive margin is not usually present in these samples. Second, immune cell densities were evaluated using tissue microarrays that do not completely represent the entire tumour. The tissue microarrays were designed to represent average immune cell infiltration of the tumours and did not specifically target SARIFA-positive regions, which could only be evaluated in a limited number of cases. Third, data on postoperative cancer treatments were not available, and the predictive value of SARIFA status needs to be evaluated in subsequent studies. Fourth, due to the low number of SARIFA-positive cases and colorectal cancer deaths in the pT1/T2 subgroup, we were unable to determine its prognostic relevance in this subgroup. Caution should be taken when interpreting SARIFA status in low pT-stage cancers, and further studies with larger cohorts are needed to clarify its value in this subgroup. Fifth, rectal cancer patients who received neoadjuvant treatment were excluded from the study. This likely resulted in an underrepresentation of rectal cancers with high pT stages and SARIFA-positivity within the cohorts. Further research is needed to investigate the prognostic significance of SARIFA status in neoadjuvant-treated patients.

## Conclusions

SARIFA status is an independent prognostic factor for cancer-specific survival and overall survival in colorectal cancer that can be reproducibly evaluated from H&E-stained tumour sections. SARIFA-positivity is associated with lower densities of T cells, CD66b^+^ granulocytes, mast cells, mature monocytic cells, and M1-like macrophages but higher densities of M2-like macrophages and immature monocytic cells in the tumours, which supports the hypothesis of an immunosuppressive tumour microenvironment in SARIFA-positive tumours.

## Supplementary information

Supplementary online material

Remark Checklist

## Data Availability

Data generated and/or analysed during this study are not publicly available. The sharing of data will require approval from relevant ethics committees and/or biobanks. Further information including the procedures to obtain and access data of Finnish Biobanks are described at https://finbb.fi/en/fingenious-service.
