# Supplementary material for: Stroma AReactive Invasion Front Areas (SARIFA), tumour immune microenvironment, and survival in colorectal cancer
Source: Br J Cancer. 2025 Mar 7;132(9):805–13. doi: 10.1038/s41416-025-02972-z (PMC12041369; doi:10.1038/s41416-025-02972-z)
Supplement: Supplementary file 1 — Supplementary online material [file 41416_2025_2972_MOESM1_ESM.pdf]

Table S1. Baseline characteristics of colorectal cancer patients according to SARIFA status in pT3 and pT4 tumors.

	Cohort 1				Cohort 2			
		SARIFA status				SARIFA status		
Characteristic	Total N	Negative	Positive	*P*	Total N	Negative	Positive	*P*
All cases	875	555 (63%)	320 (37%)		547	304 (56%)	243 (44%)	
Sex								
Female	445 (51%)	280 (50%)	165 (52%)	0.78	264 (48%)	144 (47%)	120 (49%)	0.67
Male	430 (49%)	275 (50%)	155 (48%)		283 (52%)	160 (53%)	123 (51%)	
Age (years)								
<65	239 (27%)	140 (25%)	99 (31%)	0.19	172 (31%)	90 (30%)	82 (34%)	0.49
65-75	291 (33%)	189 (34%)	102 (32%)		205 (38%)	114 (38%)	91 (37%)	
>75	345 (39%)	226 (41%)	119 (37%)		170 (31%)	100 (33%)	70 (29%)	
Year of operation								
2000-2005	265 (30 %)	166 (30%)	99 (31%)	0.67	-	-	-	0.63
2006-2010	287 (33%)	178 (32%)	109 (34%)		122 (22%)	65 (21%)	57 (23%)	
2011-2015	323 (37%)	211 (38%)	112 (35%)		158 (29%)	85 (28%)	73 (30%)	
2016-2020	-	-	-		267 (49%)	154 (51%)	113 (47%)	
Tumor location								
Proximal colon	437 (50%)	267 (48%)	170 (53%)	0.096	254 (47%)	136 (45%)	118 (49%)	0.013
Distal colon	333 (38%)	212 (38%)	121 (38%)		155 (28%)	77 (25%)	78 (32%)	
Rectum	105 (12%)	76 (14%)	29 (9%)		138 (25%)	91 (30%)	47 (19%)	
AJCC disease stage								
II	408 (47%)	327 (59%)	81 (25%)	<0.0001	253 (46%)	183 (60%)	70 (29%)	<0.0001
III	318 (36%)	158 (28%)	160 (50%)		214 (39%)	94 (31%)	120 (49%)	
IV	149 (17%)	70 (13%)	79 (25%)		80 (15%)	27 (9%)	53 (22%)	
Histological depth of subserosal invasion[A]								
>3 mm	357 (41%)	115 (21%)	242 (76%)	<0.0001	-	-	-	
≤3 mm	507 (59%)	430 (79%)	77 (24%)		-	-	-	
Tumor grade								
Low-grade	696 (80%)	465 (84%)	231 (72%)	<0.0001	453 (83%)	266 (88%)	187 (77%)	0.001
High-grade	179 (20%)	90 (16%)	89 (28%)		94 (17%)	36 (13%)	56 (23%)	
Lymphovascular invasion								
No	657 (75%)	469 (85%)	188 (59%)	<0.0001	251 (46%)	191 (63%)	60(25%)	<0.0001
Yes	218 (25%)	86 (15%)	132 (41%)		296 (54%)	113 (37%)	183 (75%)	
Tumour budding								
Grade 1	627 (72%)	450 (81%)	177 (55%)	<0.0001	348 (64%)	237 (78%)	111 (46%)	<0.0001
Grade 2	140 (16%)	68 (12%)	72 (23%)		111 (20%)	41 (13%)	70 (29%)	
Grade 3	108 (12%)	37 (7%)	71 (22%)		88 (16%)	26 (9%)	62 (26%)	
MMR status								
MMR proficient	733 (84%)	460 (83%)	273 (85%)	0.39	453 (83%)	244 (80%)	209 (86%)	0.077
MMR deficient	142 (16%)	95 (17%)	47 (15%)		94 (17%)	60 (20%)	34 (14%)	
BRAF status[B]								
Wild-type	713 (82%)	460 (83%)	253 (79%)	0.15	464 (85%)	264 (87%)	200 (83%)	0.19
Mutant	160 (18%)	93 (17%)	67 (21%)		82 (15%)	40 (13%)	42 (17%)	
Immune cell score[C]								
Low	145 (18%)	82 (16%)	63 (21%)	0.002	96 (18%)	36 (12%)	60 (25%)	0.0003
Intermediate	506 (62%)	312 (60%)	194 (65%)		321 (60%)	192 (64%)	129 (54%)	
High	163 (20%)	122 (24%)	41 (14%)		122 (23%)	73 (24%)	49 (21%)	

Abbreviations: AJCC, American Joint Committee on Cancer; MMR, mismatch repair. *P* values were calculated using the Chi-square test.

[A]Data missing for 11 cases in cohort 1 and all cases in cohort 2

[B]Data missing for 2 cases in cohort 1 and 1 case in cohort 2

[C]Data missing for 61 cases in cohort 1 and 8 cases in cohort 2

Table S2. Multivariable Cox regression models for cancer-specific survival

	Cohort 1		Cohort 2	
	Multivariable HR (95% CI)		Multivariable HR (95% CI)	
	All	pT3/pT4	All	pT3/pT4
SARIFA status				
Negative	1 (referent)	1 (referent)	1 (referent)	1 (referent)
Positive	1.75 (1.35-2.25)	1.62 (1.25-2.10)	2.09 (1.43-3.05)	2.26 (1.50-3.42)
Age				
<65	1 (referent)	1 (referent)	1 (referent)	1 (referent)
65-75	1.18 (0.87-1.58)	1.12 (0.81-1.50)	1.66 (1.10-2.52)	1.63 (1.05-2.53)
>75	1.91 (1.43-2.56)	1.83 (1.36-2.47)	2.88 (1.87-4.42)	2.77 (1.75-4.36)
Gender				
Male	1 (referent)	1 (referent)	1 (referent)	1 (referent)
Female	0.90 (0.71-1.15)	0.93 (0.73-1.20)	1.03 (0.74-1.43)	1.01 (0.70-1.44)
Year of operation				
2000-2005	1 (referent)	1 (referent)	-	-
2006-2010	0.61 (0.46-0.81)	0.56 (0.42-0.75)	1 (referent)	1 (referent)
2011-2015	0.48 (0.36-0.64)	0.45 (0.34-0.61)	1.02 (0.68-1.55)	1.08 (0.69-1.69)
2016-2020	-	-	0.63 (0.41-0.97)	0.61 (0.39-0.96)
Tumor location				
Proximal colon	1 (referent)	1 (referent)	1 (referent)	1 (referent)
Distal colon	0.92 (0.70-1.19)	0.92 (0.70-1.20)	1.15 (0.76-1.73)	1.13 (0.73-1.75)
Rectum	0.89 (0.62-1.28)	0.79 (0.53-1.17)	1.10 (0.72-1.69)	1.05 (0.66-1.68)
AJCC disease stage				
I-II	1 (referent)	1 (referent)	1 (referent)	1 (referent)
III	2.67 (1.93-3.69)	2.88 (2.03-4.08)	2.38 (1.44-3.93)	2.33 (1.31-4.14)
IV	15.2 (10.7-21.5)	14.7 (10.1-21.5)	18.8 (11.1-32.0)	19.0 (10.4-34.8)
Tumor grade				
Low-grade	1 (referent)	1 (referent)	1 (referent)	1 (referent)
High-grade	1.82 (1.35-2.45)	1.79 (1.32-2.43)	1.37 (0.90-2.07)	1.34 (0.87-2.08)
Lymphovascular invasion				
No	1 (referent)	1 (referent)	1 (referent)	1 (referent)
Yes	1.58 (1.22-2.05)	1.52 (1.17-1.98)	1.59 (1.02-2.46)	1.46 (0.90-2.36)
MMR status				
MMR proficient	1 (Referent)	1 (referent)	1 (referent)	1 (referent)
MMR deficient	0.62 (0.37-1.02)	0.68 (0.41-1.12)	0.49 (0.23-1.04)	0.48 (0.22-1.04)
BRAF mutation				
Wild-type	1 (referent)	1 (referent)	1 (referent)	1 (referent)
Mutant	1.23 (0.81-1.86)	1.18 (0.78-1.78)	1.57 (0.88-2.79)	1.77 (0.95-3.28)
Tumour budding				
Grade 1	1 (referent)	1 (referent)	1 (referent)	1 (referent)
Grade 2	1.31 (0.97-1.78)	1.32 (0.96-1.79)	1.37 (0.88-2.13)	1.35 (0.85-2.14)
Grade 3	1.20 (0.87-1.65)	1.22 (0.88-1.70)	1.41 (0.91-2.19)	1.41 (0.88-2.26)

Abbreviations: HR, hazard ratio; CI, confidence interval; MMR, mismatch repair.

Table S3. Comparison of the prognostic power of SARIFA status and tumour budding using Cox regression models for cancer-specific survival.

Variable	No. of cases	No. of events	Model 1 (Univariable) HR (95% CI)	Model 2 (multivariable) HR (95% CI)	Model 3 (multivariable) HR (95% CI)
**Cohort 1**					
SARIFA status					
Negative	752	140	1 (referent)	1 (referent)	1 (referent)
Positive	311	156	3.56 (2.83-4.48)	2.94 (2.31-3.75)	1.75 (1.35-2.25)
P			<0.0001	<0.0001	<0.0001
Tumour budding					
Grade 1	803	175	1 (referent)	1 (referent)	1 (referent)
Grade 2	148	61	2.24 (1.68-3.00)	1.71 (1.27-2.31)	1.31 (0.97-1.78)
Grade 3	112	60	3.20 (2.39-4.30)	2.06 (1.51-2.81)	1.20 (0.87-1.65)
P_{trend}			<0.0001	<0.0001	0.162
**Cohort 2**					
SARIFA status					
Negative	531	65	1 (referent)	1 (referent)	1 (referent)
Positive	240	90	3.72 (2.70-5.12)	3.01 (2.14-4.22)	2.09 (1.43-3.05)
P			<0.0001	<0.0001	0.0001
Tumour budding					
Grade 1	538	76	1 (referent)	1 (referent)	1 (referent)
Grade 2	127	35	2.13 (1.43-3.18)	1.49 (0.98-2.26)	1.37 (0.88-2.13)
Grade 3	106	44	3.68 (2.54-5.34)	2.58 (1.75-3.80)	1.41 (0.91-2.19)
P_{trend}			<0.0001	<0.0001	0.103

The analysis included patients from whom both SARIFA status and tumour budding were determined. The patients who had received preoperative treatments or died within 30 days or less after the surgery were excluded, resulting 1063 patients for cohort 1 and 771 patients for cohort 2.

Model 2: Cox proportional hazards regression model that included SARIFA status and tumour budding.

Model 3: Cox proportional hazards regression model that included SARIFA status and tumour budding and was additionally adjusted for age (<65, 65-75, <75), sex (male, female), stage (I-II, III, IV), tumour location (proximal colon, distal colon, rectum), year of operation (2000-2005, 2006-2010, 2011-2015), lymphatic or venous invasion (no, yes), grade (low-grade, high-grade), MMR status (proficient, deficient) and BRAF (wild-type, mutant).

Abbreviations: HR, hazard ratio; CI, confidence interval2

Table S4. T cells densities according to SARIFA status in cohort 2

Immune cell type	N	SARIFA status negative	SARIFA status positive	P
**Overall tumor region**				
CD3+ T cells	759	796 (521–1160)	528 (324-878)	<0.0001
CD8+ T cells	759	314 (160-533)	221 (100-430)	<0.0001
**Tumor intraepithelial region**				
CD3+ T cells	759	134 (62.0-237)	59.6 (23.8–134)	<0.0001
CD8+ T cells	759	102 (43.1-203)	40.7 (11.1-119)	<0.0001
**Tumor stromal region**				
CD3+ T cells	759	1280 (938-1730)	870 (570-1230)	<0.0001
CD8+ T cells	759	487 (280-788)	340 (176-597)	<0.0001

The numbers indicate T cell densities (1/mm^2).

Table S5. Comparison of the prognostic power of SARIFA and Immune cell score using Cox regression models for cancer-specific survival.

Variable	No. of cases	No. of events	Model 1 (Univariable) HR (95% CI)	Model 2 (multivariable) HR (95% CI)	Model 3 (multivariable) HR (95% CI)
**Cohort 1**					
SARIFA status					
Negative	694	127	1 (referent)	1 (referent)	1 (referent)
Positive	289	143	3.54 (2.79-4.50)	3.28 (2.57-4.17)	1.71 (1.32-2.23)
P			<0.0001	<0.0001	<0.0001
Immune cell score					
low	163	64	1 (referent)	1 (referent)	1 (referent)
intermediate	596	172	0.70 (0.52-0.93)	0.77 (0.57-1.02)	0.76 (0.58-1.04)
high	224	34	0.34 (0.22-0.51)	0.44 (0.29-0.67)	0.55 (0.35-0.85)
P_{trend}			<0.0001	0.0001	0.006
**Cohort 2**					
SARIFA status					
Negative	511	63	1 (referent)	1 (referent)	1 (referent)
Positive	235	88	3.66 (2.65-5.06)	3.03 (2.18-4.21)	1.86 (1.26-2.75)
P			<0.0001	<0.0001	0.002
Immune cell score					
low	113	54	1 (referent)	1 (referent)	1 (referent)
intermediate	430	78	0.31 (0.22-0.44)	0.38 (0.27-0.54)	0.71 (0.48-1.06)
high	203	19	0.16 (0.09-0.27)	0.21 (0.12-0.36)	0.54 (0.30-1.00)
P_{trend}			<0.0001	<0.0001	0.037

The analysis included patients from whom both SARIFA status and immune cell score were determined. The patients who had received preoperative treatments or died within 30 days or less after the surgery were excluded, resulting 983 patients for cohort 1 and 746 patients for cohort 2.

Model 2: Cox proportional hazards regression model that included SARIFA status and immune cell score.

Model 3: Cox proportional hazards regression model that included SARIFA status and immune cell score and was additionally adjusted for age (<65, 65-75, <75), sex (male, female), stage (I-II, III, IV), tumour location (proximal colon, distal colon, rectum), year of operation (2000-2005, 2006-2010, 2011-2015), lymphatic or venous invasion (no, yes), grade (low-grade, high-grade), tumour budding (grade 1, grade 2, grade 3), MMR status (proficient, deficient) and BRAF (wild-type, mutant).

P_{trend} values were calculated by using three ordinal categories of Immune cell score as continuous variables in univariable and multivariable Cox proportional hazard regression models.

Abbreviations: HR, hazard ratio; CI, confidence interval

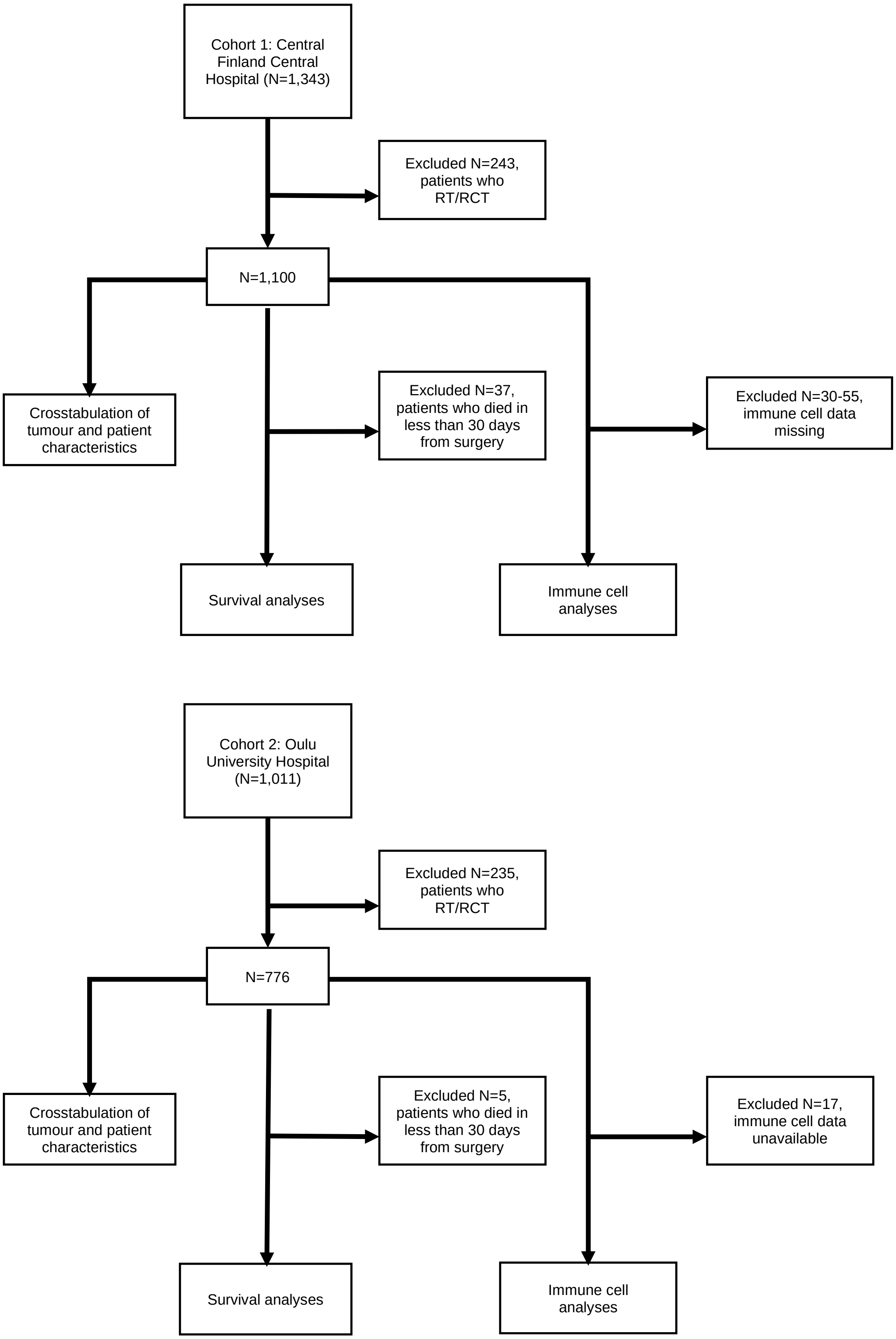

Figure S1. Flow of patients through the study.

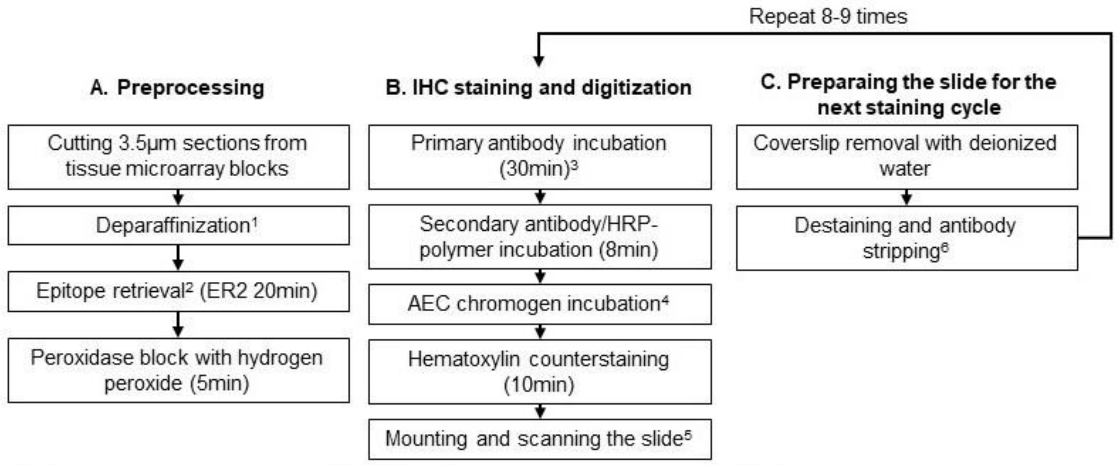

Macrophage panel	Myeloid cell panel	B cell panel
**1. CD237** [E1L3N], #13684S, Cell Signaling, 1:100	**1. CEACAM8** [G10F5], #305102, BioLegend, 1:100	**1. CD20** [L26], #Mob004, Diagnostic Biosystems, 1:100
**2. CD3** [LN10], #PA055, Leica, ER1 60min, 1:1	**2. FCGR3** [D1N9L], #24316S, Cell Signaling, ER1 30min, 1:70	**2. CD79A** [SP18], #RM-9118, Thermo Scientific, ER1 60min, 1:600
**3. PDCD1** [SP269], #ab2277681, Abcam, ER1 30min, 1:40	**3. CD14** [D7A2T], #75181S, Cell Signaling, ER1 60min, 1:70	**3. IRF4** [MUM1p], #M7259, Dako, ER1 60min, 1:200
**4. CD163** [10D6], #MS-1103, Thermo Scientific, ER1 60min, 1:100	**4. HLA-DR** [TAL1B5], #sc-53319, Santa Cruz, ER1 60min, 1:4000	**4. IgG1** [RM117], #31-1019-00, RevMAb, ER1 60min, 1:4000
**5. CD86** [E2G8P], #91882S, Cell Signaling, ER1 30min, 1:50	**5. ITGAM** [D6X1N], #49420S, Cell Signaling, ER1 30min, 1:60	**5. HLA-DR** [TAL1B5], #sc-53319, Santa Cruz, ER1 60min, 1:4000
**6. HLA-DR** [TAL1B5], #sc-53319, Santa Cruz, ER1 30min, 1:4000	**6. CD33** [SP266], #ab199432, Abcam, ER1 60min, 1:500	**6. IgG2** [EPR4418], #ab134050, Abcam, ER1 60min, 1:40 000
**7. MRC1** [E2L9N], #91992S, Cell Signaling, ER1 30min, 1:500	**7. TPSAB1** [AA1], #sc-59587, Santa Cruz, ER1 60min, 1:500	**7. IgG4** [HP6025], #605-900, Thermo Scientificm ER1 60min, 1:500
**8. CD68** [KP1], #916104, BioLegend, ER1 30min, 1:40 000	**8. ARG1** [EPR6672(B)], "ab133543, Abcam, ER1 30min, 1:500	**8. KRT** [BS5], #BSH-7124-1, BioSite Histo, ER1 60min, 1:400
**9. KRT** [BS5], #BSH-7124-1, BioSite Histo, ER1 60min, 1:400	**9. IDO** [EPR20374], #ab211017, Abcam, ER1 60min, 1:500	
	**10. KRT** [BS5], #BSH-7124-1, BioSite Histo, ER1 60min, 1:400	

Figure S2. Multiplex immunohistochemistry protocols including the details of primary antibodies used in the three assays.
Abbreviations: IHC, immunohistochemistry; AEC, 3-Amino-9-ethylcarbazole
[1] Dewax solution (AR9222, Leica Biosystems), 30min, 60–72 ˚C.
[2] Epitope retrieval done with BOND epitope retrieval solution 2 (EDTA based, pH 9, AR9640, Leica Biosystems) with 20min heating time.
[3] Primary antibodies (name, clone, catalogue number and manufacturer) together with the selected antigen retrieval conditions and used dilutions are listed in the staining order.
[4] AEC + high sensitivity substrate (K3469, Dako).
[5] VectaMount AQ Aqueous Mounting Medium (H-5501, Vector Laboratories), digitized with a 20x objective using NanoZoomer XR (Hamamatsu) slide scanner.
[6] The sections were destained with ethanol and antibody stripping was done with heat-induced epitope retrieval using BOND epitope retrieval solution 1 (citrate based, pH 6, AR9961, Leica Biosystems) or 2. Suitable epitope retrieval conditions were optimized for each antibody.

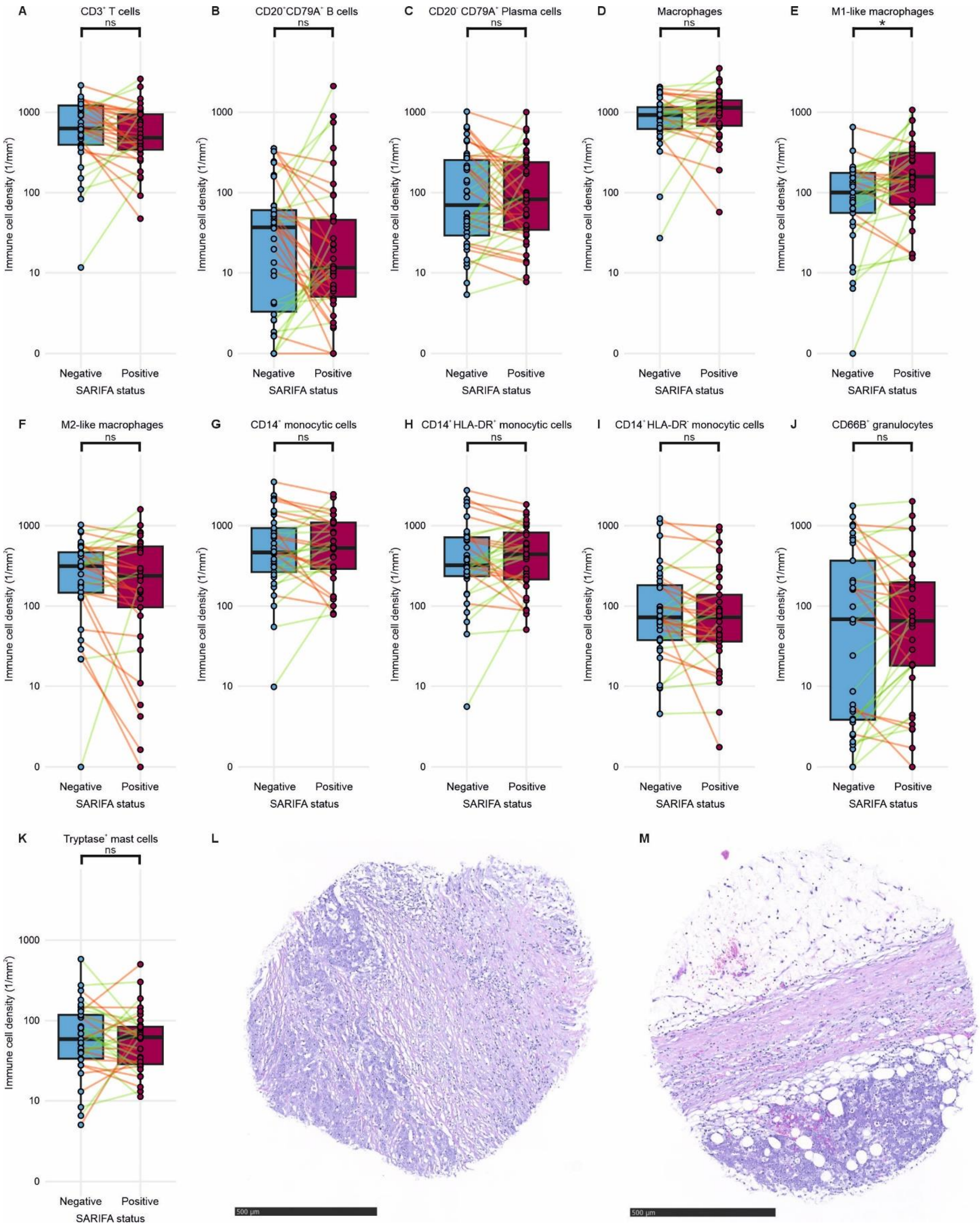

Figure S3. Immune cell densities in cases with discordant SARIFA status in invasive margin cores of tissue microarrays. (A-K) Boxplots showing immune cell densities in paired SARIFA-positive and SARIFA-negative invasive margin cores. Individual cases are presented by connecting lines: green lines indicate cases where the SARIFA-positive core had higher immune cell density, while red lines indicate cases where the SARIFA-negative core had higher immune cell density. Analyses were based on tissue microarray based immune cell data (cohort 1) In total, 49 cases exhibited discordant SARIFA status in the tissue microarrays. Cases lacking immune cell data from both SARIFA-negative and SARIFA-positive cores were excluded, resulting in the following sample sizes: N=33 cases for CD3$^+$ T cells, macrophages, M1-like macrophages, M2-like macrophages, CD14$^+$ monocytic cells, CD14$^+$HLA-DR$^+$ mature monocytic cells, CD14$^+$HLA-DR$^-$ immature monocytic cells, CD66B$^+$ granulocytes, and tryptase$^+$ mast cells; and N=38 cases for

$CD20^+CD79A^+$ B cells and $CD20^-CD79A^+$ plasma cells. Statistical significance is indicated as ns ($p>0.05$), * ($p<0.05$). P values were calculated with Wicoxon signed-rank test. (L, M) Example images illustrating discordant SARIFA status, with one SARIFA-negative core (L) and one SARIFA-positive core (M). Scale bars are 500 µm.
